# Influence of discrete fracture network on the performance of enhanced geothermal system considering thermal-hydraulic-mechanical multi-physical field coupling

**DOI:** 10.1371/journal.pone.0320015

**Published:** 2025-04-23

**Authors:** Haiyang Jiang, He Huang, Liangliang Guo, Yanling Cao, Yanchun Hu, Su Cui, Bo Wu, Fengxin Kang, Ling Zhang, Meng Shi, Xueyu Zhou

**Affiliations:** 1 No.1 Institute of Geology and Mineral Resource Exploration of Shandong Province, Jinan, China; 2 Key Laboratory of Groundwater Resources and Environment, Ministry of Education, Jilin University, Changchun, China; 3 River Basin Planning & Policy Research Center of Songliao River Water Resources Commission, Changchun, China; 4 College of Water Resources Science and Engineering, Taiyuan University of Technology, Taiyuan, China; 5 Engineering Research Center of Geothermal Resources Development Technology and Equipment, Ministry of Education, Jilin University, Changchun, China; 6 College of Marine Geo-science, Ocean University of China, Qingdao, China; 7 College of Earth Science and Engineering, Shandong University of Science and Technology, Qingdao, China; 8 Shandong Geology and Mining New Energy Co., Ltd, Jinan, China; 9 Shandong No.3 Exploration Institute of Geology and Mineral Resources, YanTai, China; China University of Mining and Technology, CHINA

## Abstract

The long-term circulation of cold water within the deep heat reservoir in enhanced geothermal system (EGS) involves dynamic evolution of thermal-hydraulic-mechanical (THM) fields. The characteristics of discrete fracture network (DFN) in the reservoir also affect the thermal performance of EGS. In this paper, the influence of the DFN on the performance of EGS considering THM multi-physical field couple was numerically investigated. The effect of water property change and cold tension stress with temperature was considered. The influence of DFN characteristics (fracture density, fracture aperture, angle between fracture and well connection line and fracture pattern) was discussed. Results indicate that the effect of considering water property change on the Darcy velocity is larger than considering the stress field. Considering water property change and stress field both lead to a larger Darcy velocity of the fracture water and make the temperature field drop faster. With the increase of fracture density, the production Darcy’s velocity increases gradually and the difference in thermal recovery efficiency is significant. Fracture aperture has a greater influence on the temperature field of the whole DFN reservoir than fracture density. The larger the fracture aperture, it will take away the heat energy from the heat reservoir faster. The smaller the angle between fracture and well connection line, the smaller the flow resistance of the working fluid, and the larger the equivalent permeability of the reservoir DFN system. The differences in the seepage field are small for the same range of statistical characteristics and parameter values for the DFN patterns. The DFN system with uniform fracture distribution should be selected for a better EGS reservoir, and the angle between the direction of the main fracture group and the direction of the well connection line should be at a certain acute angle.

## Introduction

Geothermal energy is a renewable and clean energy with abundant reserves and wide distribution [1]. Geothermal resources can be divided into hydrothermal and hot dry rock (HDR) geothermal resources. HDR geothermal resources refers to the high-temperature heat energy stored in the dense rock mass between 3 ~ 10 km underground [2,3]. HDR reserves are huge and suitable for base-load power supply, independent of surface climate and environmentally friendly. Therefore, the development of HDR geothermal energy plays an important role in safeguarding the energy structure and reducing carbon emissions.

Currently, the main way to develop HDR geothermal resources is to establish enhanced geothermal systems (EGS) [4]. EGS involves three main process. First, the cold water was injected into deep HDR reservoir with multiple discrete fracture network (DFN). The cold water would then continuously extract the heat stored in the wall rock during long term circulation. Finally, the heated water was produced to the surface for power generation. The process of cold water circulation involves multidisciplinary issues such as seepage, heat transfer, rock deformation, etc. After the cold water is injected into the heat reservoir, the temperature field distribution becomes extremely heterogeneous. Due to the temperature difference between water and rock constituents, thermal stresses can be generated in areas with large temperature gradients. Thermal stresses in turn change the distribution of stress field and cause rock deformation, which could affect the distribution of the seepage field. Changes in seepage pressure and flow velocity also could change the distribution of the stress field and temperature field. On the other hand, if the change in the properties of water such as kinetic viscosity and density with temperature were considered, the water property in different temperature regions would affect the distribution of seepage pressure and Darcy velocity. Therefore, the multi-physical field coupling of thermal-hydraulic-mechanical (THM) field needs to be considered in the process of thermal energy extraction from EGS.

Numerical simulation is the main technique to study multi-physics coupling of EGS. Lei et al. developed a coupled HM model that incorporates shear slip and the poroelastic response of the rock matrix. It is concluded that the connectivity of the fracture network has a strong control on the rock damage [5]. Guo et al. established a coupled TH model and carried out a sensitivity analysis of reservoir parameters and operational parameters [6]. Zhou et al. proposed a THM coupled model of geothermal reservoir including regular connected fractures and irregular discrete fractures [7]. The fracture characteristic, coupling conditions and model parameters would affect the thermal performance. Changes in temperature create thermal stresses in the rock, which in turn leads to expanding micro-crack in the rock. Song et al. developed a fully coupled THMC model for the production of an EGS based on fracture aperture alternation [8]. The mechanical effect plays a major role near the injection well and in a short term. The chemical effect is dominated far from the injection well and its time scale of action is long-term. Ebrahimi et al. developed a fully-coupled THM approach to simulate the injection process in EGS [9]. It was found that poroelastic effects are prevalent in the early phases, whereas thermoelastic effects were discovered to be dominating in the long-term injection stages. Sun et al. established a THM coupled model consider fracture compression closure, shear dilation, and thermally-induced fracture opening [10]. Guo et al. established a coupled THM model to study the factors affecting fracture open under thermal stress [11]. Zhang et al. pointed out that during EGS fracturing, the temperature difference between hot rock and cold water could produce thermal tensile stresses, which can reduce the rock fracturing pressure and form branching fractures [12]. Shi et al. studied the rock damage evolution in the production process of EGS considering thermal-hydrological-mechanical and damage [13]. Damage will cause changes such as reduced Young’s modulus (negative feedback) and increased permeability (positive feedback) of the rock matrix, which in turn affect the damage evolution. Song et al. investigated the magnitudes of multi-physics effects on geothermal reservoir characteristics during the production of EGS [4]. They found that mechanical effects lead to an increase in fracture aperture, while chemical effects contribute to its reduction under underbalanced injection conditions. Strong mechanical-chemical coupling effects, exhibiting a negative correlation with chemical effects, conversely result in a diminished fracture aperture. Besides, the rock mechanical properties and constitutive relations would change with temperature. The mechanical properties of granite increase until a certain temperature threshold and then decrease with increasing temperature. The brittle properties of granites at low levels of stress and room temperature diminish with increasing stress and temperature.

Discrete fracture network (DFN) in EGS reservoir is the main conduit for the transfer of mass and heat for the working fluid. The statistical information of fractures around well is generally obtained by logging. The distribution information function of fractures is obtained after data processing and analysis. The DFN system is generated based on the information of fracture spatial distribution, length and aperture distribution function. Therefore, it is more practical to establish a DFN reservoir model. Xiong et al. developed a three-dimensional coupled TH model based on Galerkin’s finite element method for DFNs, which used a simplified heat transport model based on the localized thermal nonequilibrium theory [14]. Luo et al. concluded that fracture surface roughness has a significant effect on the fluid flow and heat transfer behavior of fractures [15]. Sun et al. developed a coupled THM mathematical model and a three-dimensional numerical model of randomly fractures [16]. Mahmoodpour et al. concluded that for a given DFN system, its fracture aperture, and rock matrix permeability are the most influential parameters in controlling the thermal breakthrough time and overall energy recovery [17]. Zhang et al. proposed a fully THMC-coupled model based on the extended finite element method (XFEM) is proposed and verified [18]. The evolution of heat production and environmental benefits of an EGS over 30 years are presented. Li et al. calculated various connectivity parameters, including the number and length of intersection lines between fracture surfaces, to analyze the dominant flow direction in three-dimensional fracture networks [19]. Lei et al. conducted a THM simulation on the impact of fracture network connectivity on the production performance of a multi-fracture EGS [20]. They confirmed that the connected area ratio is an effective indicator of the connectivity level of the fracture network. Fluid flow and heat exchange predominantly take place within the interconnected area of fractures. EGS is a multi-fracture system with natural, thermally stimulated, and hydraulic fractures. The characteristics of the fracture network, including connectivity, fracture orientation, fracture numbers, crossing angle of fractures, and network geometry, significantly impact flow and heat transfer, affecting the EGS’s performance. Existing evaluation methods to quantify the impact of fracture network characteristics on the production performance of multi-fracture EGSs is lacking.

**Fig 1:**
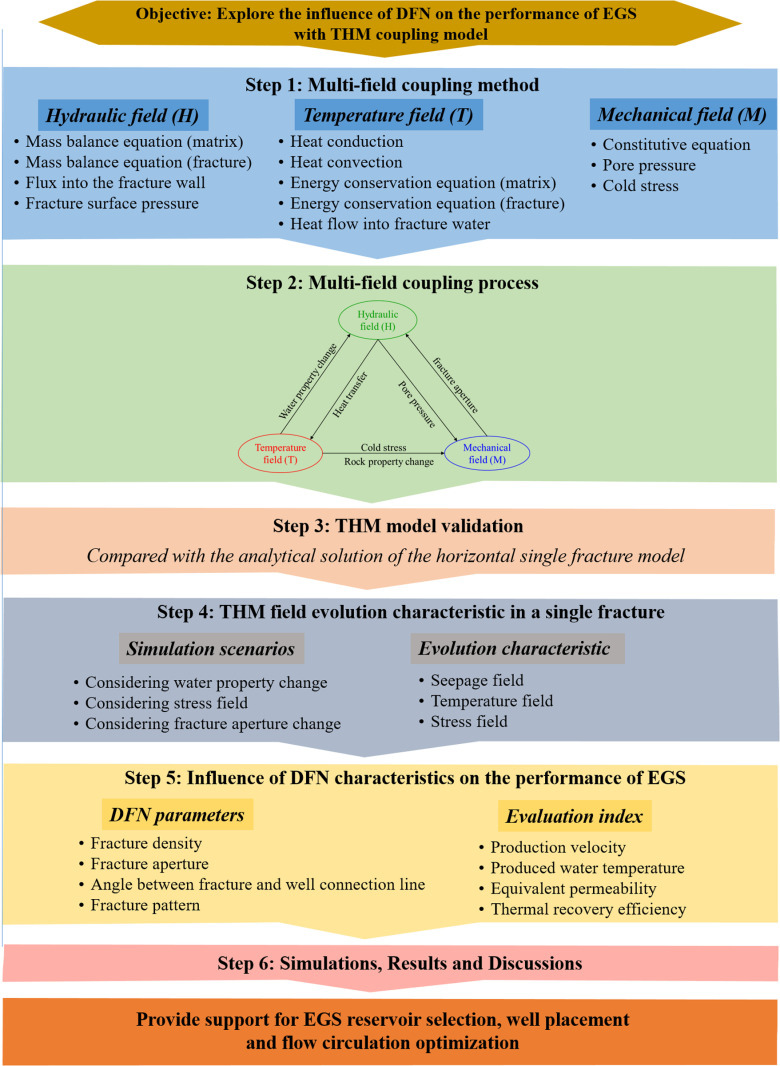
Methodological flowchart.

Currently, the accurate modeling of THM coupling in the reservoir still has a limitation, while the water property change and fracture aperture change of the DFN-EGS reservoir are rarely studied. In this paper, a coupled THM multi-physical field model of EGS is established and proved with the analytical solution. The variation of water density, thermal conductivity and specific heat capacity with temperature is considered. The influence of tension stress caused by temperature difference between injected cold water and bedrock is discussed. The effect of DFN characteristics on thermal performance of EGS was investigated. Production Darcy’s velocity, produced water temperature, equivalent permeability and thermal recovery efficiency, were used to evaluate the thermal performance of EGS reservoir. The research results can provide a support for the reservoir selection and stimulation of EGS projects. The methodological flowchart is illustrated in Fig 1.

## Multi-field coupling method

### Hydraulic field (H)

When fracture aperture is between 0.01 and 1 mm, the calculated seepage rate of fracture water is between 10^-5^ and 10^-3^ m/s. The calculated Reynolds is below 10. Therefore, the seepage state of fracture water within the HDR is laminar in accordance with Darcy’s law. The seepage mass balance equation in rock matrix is:


$$\begin{array}{c} S_{s}\frac{\partial P_{s}}{\partial t}+\nabla •\left(-\frac{k}{\mu_{w}}\nabla P_{s}\right)=-\frac{\partial e}{\partial t} \end{array}$$ (1)

where *S*_*s*_ is the unit water storage capacity of the rock matrix. *P*_*s*_ is the pressure of the rock matrix. *e* is the volumetric strain of the rock mass. *k* is the rock permeability. *μ*_*w*_ is the dynamic viscosity of water.

The seepage mass balance equation in fracture is:


$$\begin{array}{c} d•S_{f}\frac{\partial P_{f}}{\partial t}+d•\nabla_{T}•\left(-\frac{k_{f}}{\mu_{w}}\nabla_{T}P_{f}\right)=f_{up}^{f}+f_{bottom}^{f} \end{array}$$ (2)

where *d* is the width of the fracture. *S*_*f*_ is the unit water storage volume of the fracture. *P*_*f*_ is the pressure of the fracture. *k*_*f*_ is the fracture permeability. $\nabla_{T}$ is the derivation along the tangent direction of the fracture. $f_{up}^{f}$ and $f_{bottom}^{f}$ are the amount of fluid that flows into the fracture unit along the fracture’s normal direction via the upper surface and the lower surface, respectively.

The fracture unit is considered as the boundary of the rock matrix. The flux into the fracture through the upper and lower surfaces are shown:


$$\begin{array}{c} f_{up}^{f}=-\frac{k}{\mu_{w}}\frac{\partial P_{up}}{\partial n_{up}} \end{array}$$ (3)

$$\begin{array}{c} f_{bottom}^{f}=-\frac{k}{\mu_{w}}\frac{\partial P_{bottom}}{\partial n_{bottom}} \end{array}$$ (4)

where *P*_*up*_ and *P*_*bottom*_ denote the pressure of the rock on the upper and lower surfaces of the fracture, respectively.

The fracture surface pressures are all continuous, so


$$\begin{array}{c} P_{up}=P_{bottom}=P_{f} \end{array}$$ (5)

### Temperature field (T)

The Fourier’s law was used to quantitatively describe the heat transfer process. The formula is as follows:


$$\begin{array}{c} \phi =-\lambda_{T}A\frac{\partial T}{\partial n} \end{array}$$ (6)

where *ϕ* is the heat flux. $\lambda_{\text{T}}$ is the thermal conductivity. *A* is the cross-sectional area. ∂T/∂n is the temperature gradient perpendicular to the direction of that cross-section.

Newton’s cooling equation was used to quantitatively describe the thermal convection process. The formula is as follows:


$$\begin{array}{c} \phi =hA\Delta T \end{array}$$ (7)

where *h* is the convective coefficient. ΔT is the temperature difference.

The porosity of the dense rock matrix is generally about 0.05% and the permeability is about 5 × 10^-20^ to 2 × 10^-18^m^2^ [21]. Thus, water seepage rate in dense rock matrix is very small. The heat exchange between the rock matrix and the pore water is considered as local heat balance. Besides, Chen et al. studied the heat transfer through a single fracture with a width of 1 mm [22]. The results show that the calculated temperature are almost the same under the local thermal equilibrium and disequilibrium methods. Therefore, in this paper, it is assumed that the temperature of the fracture wall and the fracture water are the same. The energy conservation equation in the rock matrix is:


$$\begin{array}{c} C\frac{dT}{dt}+\nabla •\left(-\lambda_{eq}\nabla T\right)+C_{w}\left(-\frac{k}{\mu_{w}}\nabla P\right)•\nabla T=0 \end{array}$$ (8)

where *C* is the equivalent volumetric heat capacity of the rock matrix. *λ*_*eq*_ is the equivalent heat transfer coefficient of the rock matrix. *C*_*w*_ is the volumetric heat capacity of water.

The energy conservation equation within the fracture is:


$$\begin{array}{c} d\frac{\partial C_{f}T_{f}}{\partial t}+d\rho_{w}c_{w}\vec{u_{w}}•\nabla_{T}T_{f}-d\nabla_{T}•\left(\lambda_{f}\nabla_{T}T_{f}\right)=f_{up}^{e}+f_{bottom}^{e} \end{array}$$ (9)

where *C*_*f*_ is the equivalent volumetric heat capacity of the fracture water. *λ*_*f*_ is the equivalent heat transfer coefficient of the fracture water. ${\vec{u}}_{w}$ is the Darcy velocity of the of the fracture water. $f_{up}^{e}$ and $f_{bottom}^{e}$ are the energies transferred into the fracture through the upper and lower surfaces of the fracture.

When the fracture unit is considered as the boundary of the rock matrix. The expressions for the heat flow into the fracture water through the upper and lower surfaces are shown:


$$\begin{array}{c} f_{up}^{e}=\rho_{w}c_{w}\left(-\frac{k}{\mu_{w}}\frac{\partial P_{up}}{\partial n_{up}}\right)T_{up}-\lambda_{eq}\frac{\partial T_{up}}{\partial n_{up}} \end{array}$$ (10)

$$\begin{array}{c} f_{bottom}^{e}=\rho_{w}c_{w}\left(-\frac{k}{\mu_{w}}\frac{\partial P_{bottom}}{\partial n_{bottom}}\right)T_{bottom}-\lambda_{eq}\frac{\partial T_{bottom}}{\partial n_{bottom}} \end{array}$$ (11)

### Mechanical field (M)

Granite is considered to be a porous medium elastomer. The constitutive equation for granite considering the combined effect of pore pressure and thermal stress is


$$\begin{array}{c} \sigma_{ij,j}+F_{i}=0 \end{array}$$ (12)

$$\begin{array}{c} \mu u_{i,jj}+\left(\lambda +\mu \right)u_{j,ji}-\alpha_{B}p_{,i}-\beta_{T}T_{s,i}+F_{i}=0 \end{array}$$ (13)

where


$$\begin{array}{c} \mu =\frac{E}{2\left(1+\upsilon \right)} \end{array}$$ (14)

$$\begin{array}{c} \lambda =\frac{E\upsilon }{\left(1+\upsilon \right)\left(1-2\upsilon \right)} \end{array}$$ (15)

$$\begin{array}{c} \beta_{T}=\alpha_{T}\frac{E}{1-2\upsilon } \end{array}$$ (16)

where $\sigma_{ij}$ is the second-order tensor component of stress. *u* denotes displacement. *u*_i,jj_ and *u*_j,ji_ are the second-order tensor component of displacement. *F*_i_ represents body force. *μ* (second-order Lamet constant) represents the shear modulus of the material. *λ* is a first-order Lamet constant that represents the compressibility of a material. *E* denotes elastic modulus. *v* represents Poisson’s ratio. *p* represents seepage pressure. $\alpha_{B}$ is the Biot-Willis coefficient, $\alpha_{B}$ ≤ 1. *T*_*s*_ represents the temperature of the rock mass. $\alpha_{T}$ represents the coefficient of thermal expansion. $\alpha_{B}p_{,i}$ and $\beta_{T}T_{s,i}$ represent pore pressure and thermal stress terms, respectively.

### Multi-field coupling process

#### (1) Seepage and heat transfer.

The effect of seepage on heat transfer is manifested in the effect on thermal convection. The values of pressure and seepage velocity used in the calculation of transferred heat by thermal convection are the values of pressure and Darcy flow velocity in the seepage field. The effect of heat transfer on seepage is manifested in changes in water properties. When temperature changes, the water density, specific heat capacity, thermal conductivity and dynamic viscosity could also changes [23].

When temperature is between 293.15K ≤  T ≤  373.15K, the relationship between water density and temperature is as follows:


$$\rho_{\text{water}}=1.03E-5\cdot T^{3}-1.34E-2\cdot T^{2}+4.97\cdot T+432.26$$ (17)

When temperature is between 293.15K ≤  T ≤ 553.75 K, the relationship between water specific heat capacity and temperature is as follows:


$$Cp_{\text{water}}=-5.38E-4\cdot T^{3}+0.31\cdot T^{2}-80.41\cdot T+1.20E4$$ (18)

When temperature is between 293.15K ≤  T ≤ 553.75 K, the relationship between water thermal conductivity and temperature is as follows:


$$k_{\text{water}}=7.98E-9\cdot T^{3}-1.58E-5\cdot T^{3}+8.95E-3\cdot T-0.87$$ (19)

The relationship between water dynamic viscosity and temperature is as follows:


$$eta_{\text{water}}=4.65E-7\cdot T^{3}+1.36E-4\cdot T^{2}-2.12E-2\cdot T+1.38(293.15K\leq T\leq 413.75K）$$ (20)

$$eta_{\text{water}}=-2.40E-11\cdot T^{3}+3.86E-8\cdot T^{2}-2.11E-5\cdot T+0.004(413.15K<T\leq 553.75K）$$ (21)

#### (2) Seepage and stress.

The effect of seepage on stress distribution is manifested in the deformation of the rock matrix caused by changes in pore pressure. The effect of stress distribution on seepage is manifested in changes in fracture permeability due to fracture closure and opening. When the high temperature fracture wall is in contact with cold water, the tensile stress will be generated near the fracture wall due to the shrinkage of rock particles. Tensile stresses would cause fractures to expand and then make water seepage rate become larger. Because fractures within HDR reservoirs are the main conduits for mass and heat transfer, the effect of fracture wall stress changes on permeability cannot be ignored. The following exponential empirical formula was used to calculate the relationship between fracture stress and permeability coefficient [24]:


$$\begin{array}{c} k_{f}=k_{0,f}\exp(-\alpha \sigma_{n}^{'}) \end{array}$$ (22)

where $k_{0,f}$ represents the initial permeability of the matrix. *α* represents the influence coefficient, which is related to the state of the fracture in the rock mass. $\sigma_{n}^{'}$ denotes the normal stress on the fracture wall.

#### (3) Heat transfer and stress.

The effect of heat transfer on stress distribution is manifested in thermal stresses due to uneven temperature distribution. In this paper, the thermal stress action term was added to the rock eigenstructure equation. The effect of stress distribution on heat transfer is consistent with the effect of stress distribution on seepage. Rock stresses change the porosity and permeability, thus affecting convective heat transfer.

The above mentioned partial differential equations for THM fields were compiled into COMSOL Multiphysics software for next numerical simulation.

### Model validation

**Fig 2:**
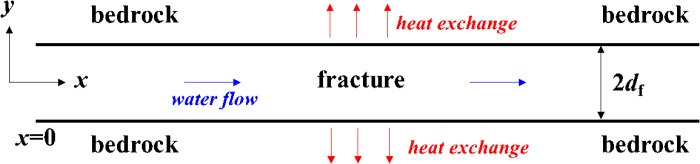
Concept diagram of single fracture model.

In order to verify the accuracy of the above multi-physics coupling numerical model, the model is compared with the analytical solution of the horizontal single fracture model (Fig 2). Low-temperature water enters from the fracture left, then exchanges heat with the upper and lower bedrocks, and finally exits at the fracture right end carrying heat away.

Cheng et al. combined the factors of heat conduction and heat convection in the above single fracture model, thus giving an expression for the temperature at different locations [25]. The equation is as follows:


$$\begin{array}{c} \begin{array}{rr} T-T_{0}= & \frac{2\left(T_{1}-T_{0}\right)}{\sqrt{\pi }}\cdot \text{exp}\left(\frac{xv}{2D}\right)\cdot \int_{\frac{x}{2\sqrt{Dt}}}^{\infty }{\text{e}}^{-\sigma^{2}-{\left(\frac{xv}{4D_{\sigma }}\right)}^{2}}\\ & \text{erfc}\left[\frac{x^{2}h^{\prime }\sqrt{D^{\prime }}}{8DH\sigma^{2}}{\left(t-\frac{x^{2}}{4D\sigma^{2}}\right)}^{-0.5}\right]\text{d}\sigma \end{array} \end{array}$$ (23)

**Fig 3:**
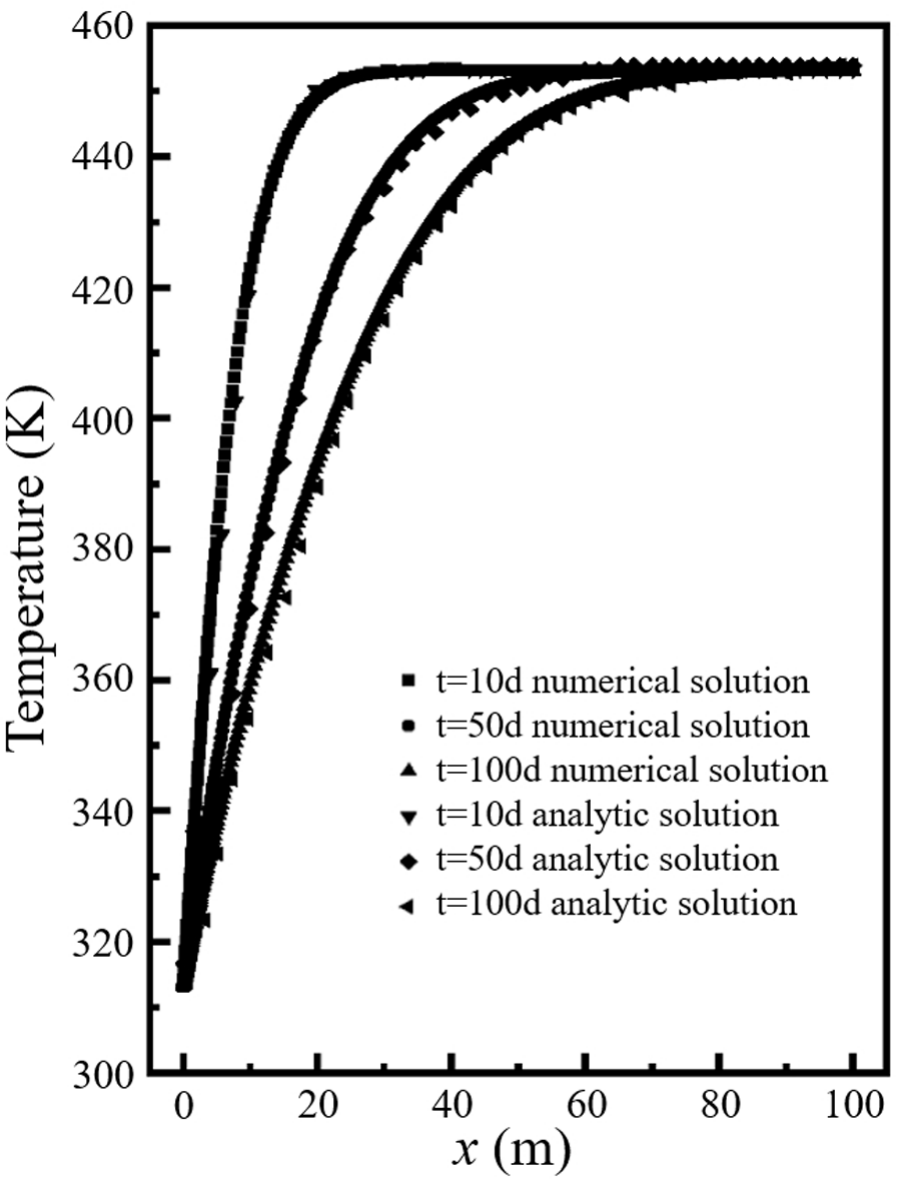
Variation of analytical and numerical solutions of temperature at different locations at three moments.

**Fig 4:**
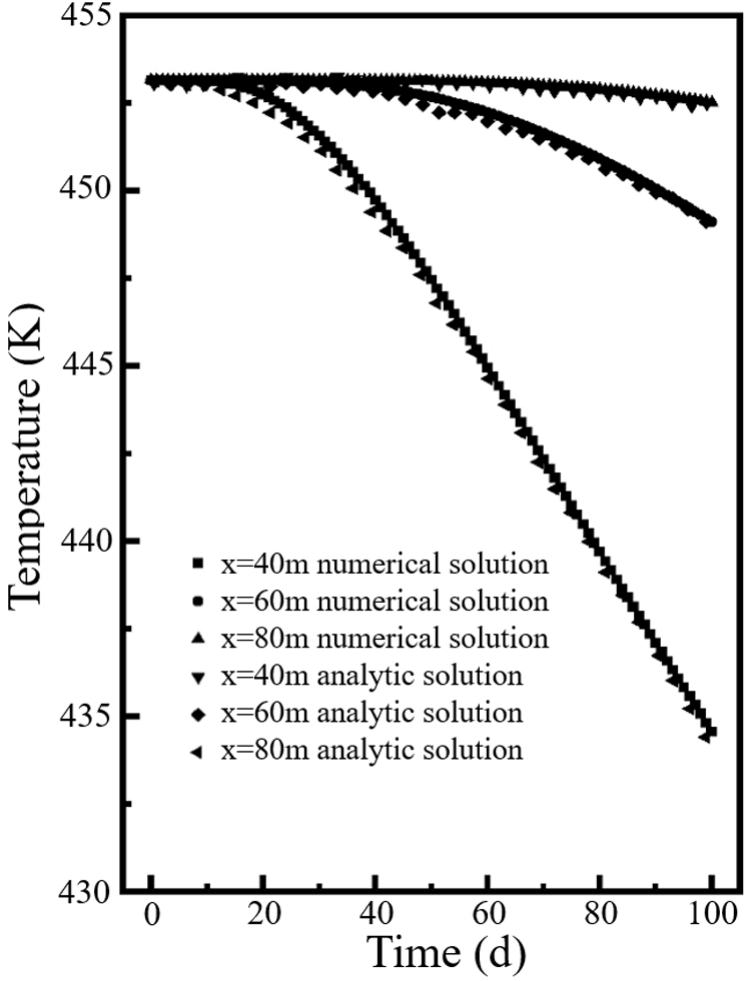
Analytical and numerical solutions of the temperature at different locations.

Because the Eq. (23) contains an infinite integral and an error function, it is difficult to obtain a definite solution. Shao used numerical integration to give an analytical solution to the Eq. (23) [26]. Thus, the numerical model established in this paper is consistent with the modeling conditions in Ref. [26] for comparison. Fig 3 shows the variation of analytical and numerical solutions of temperature at different locations at three moments *t* = 10d, 50d and 100d. Fig 4 shows the analytical and numerical solutions of the temperature at different locations of *x* = 40m, 60m and 80m. It can be seen that the analytical solution of the numerical solution agrees very well with that of Ref. [26]. It proves the reliability of our numerical model.

## Multi-physics field evolution characteristic in a single fracture

### General consideration

The evolution characteristics of THM fields in single fracture is the basis of the DFN thermal reservoir. Firstly, the THM coupling numerical model of single fracture is established, and the evolution characteristics of different physical fields are studied. Secondly, the influence of the water properties (density, specific heat capacity, thermal conductivity and dynamic viscosity) with temperature is studied. Then, the influence of stress field with temperature is discussed. The simulation scenarios include three cases:

Case a: Stress field is not considered, and the change of the water properties is not taken into account.
Case b: Stress field is not considered, and the change of the water properties is taken into account.
Case c: Consider stress field and consider the change of the water properties.

### Model setup

**Fig 5:**
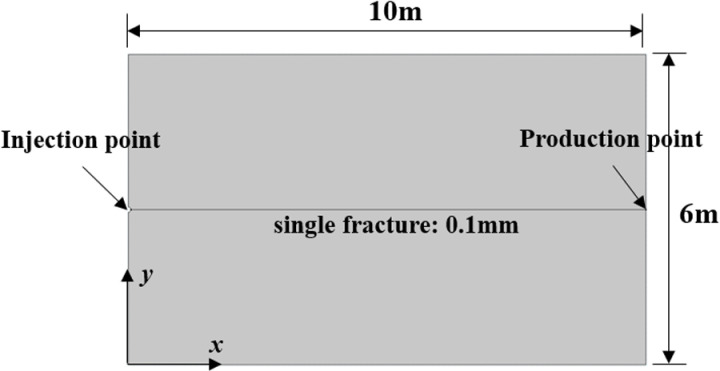
2D geometric model of single fracture.

The 2D geometric model is a rectangle with a length of 10m and a width of 6m. The single fracture is at the location of *y* = 3m with a width of 0.1mm. At the left end (*x* = 0m, *y* = 3m), a semicircle with a radius of 0.05m is set up to simulate the injection well (Fig 5). The bedrock is granite.

For the boundary conditions, the injection well is set to constant pressure of 1 ×  10^6^ Pa and a fixed temperature of 20°C. The right end is set to constant pressure of 3 ×  10^5^ Pa. The upper and lower boundaries are set to no flow and heat exchange. Semi-circumference at injection well is set with roll support. Production well, top and bottom boundaries are set to fixed constraints.

The initial pore pressure of the bedrock is 3 ×  10^5^ Pa. The initial bedrock temperature is 180°C. The initial stress field are no displacement and no structural velocity. A total of 16272 mesh units and 478 boundary units were divided.

The granite parameters at a depth of 4000m in Ref. [27] are used as the material parameters of this model, as shown in Table 1.

**Table 1:** Material parameters of granite used in the model.

Parameters	Value
Density	2.608 g/cm^3^
Porosity	1.46%
Permeability	1.00 × 10^–18^ m^2^
Thermal conductivity	2.788 W/(m ∙ ℃)
Specific heat capacity	770 J/(kg ∙ ℃)
Elastic modulus	32.17 GPa
Poisson’s ratio	0.28
Coefficient of thermal expansion	3.60 × 10-6 ℃^-1^

## Simulation results and discussions

### Variation of seepage field

**Fig 6:**
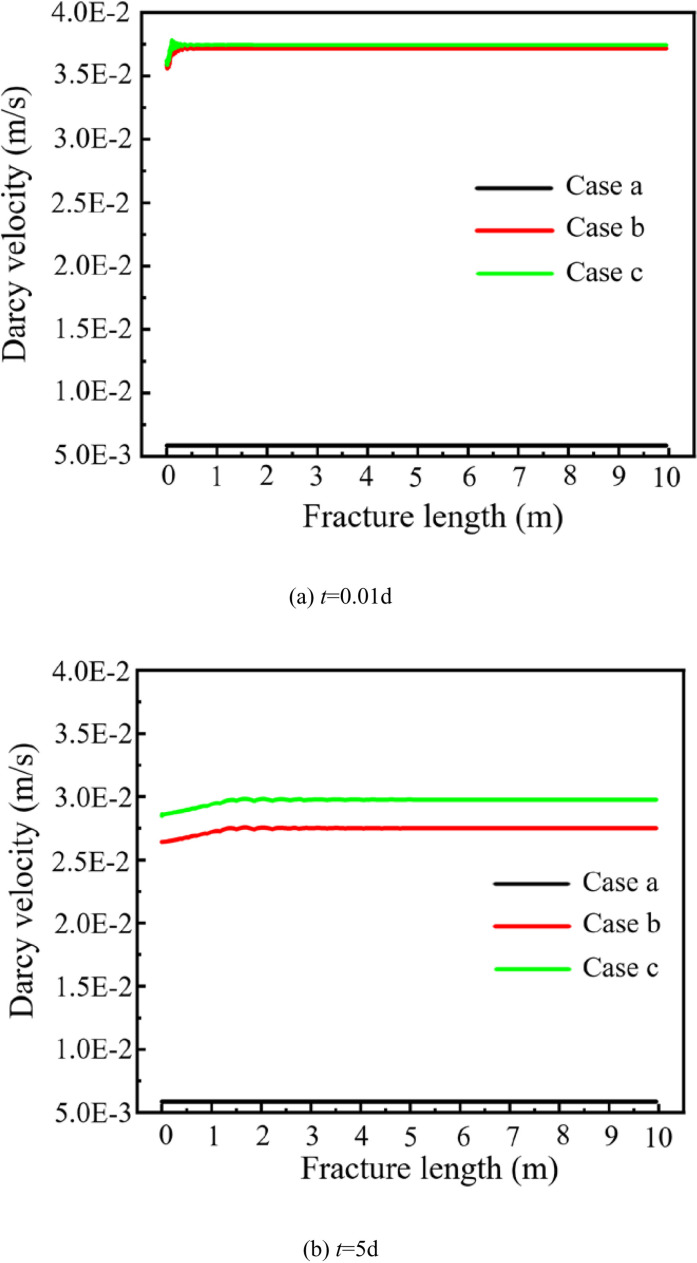
Variation of Darcy’s velocity of fracture water with time under different cases.

Fig 6 shows the variation of Darcy’s velocity of in the fracture water with time under different cases.

At the time node of *t* = 0.01d; the Darcy velocities at the production point in Case a, b and c are 0.00586 m/s, 0.03715 m/s and 0.03744 m/s, respectively. The Darcy velocity at the production point in Case b is 6.34 times that of Case a, and at the production point in Case c is 6.39 times that of Case a.

At the time node of *t* = 5d, the Darcy velocities at the production point in Case a, b and c are 0.00586 m/s, 0.02750 m/s and 0.02977 m/s, respectively. The Darcy velocity at the production point in Case b is 4.69 times that of Case a, and at the production point in Case c is 5.08 times that of Case a.

In Case a, the Darcy velocity remains constant with time. In Case b, the Darcy velocity at *t* = 5d decreases by 25.98% compared to that at *t* = 0.01d. In Case c, the Darcy velocity at *t* = 5d decreases by 20.49% compared to that at *t* = 0.01d. It can be seen that the effect of considering the change of water property on the Darcy velocity is larger than considering the stress field. Considering the change of the water property and considering the stress field both lead to a larger Darcy velocity of the fracture water. This is because when the temperature increases from 20°C to 180°C, the water dynamic viscosity decreases from 1 ×  10^-3^ Pas to 1.53 ×  10^-4^ Pas, the water density decreases from 998 kg/m^3^ to 958 kg/m^3^, and the permeability increased from 8.15 ×  10^-3^ m/s to 5.11 ×  10^-2^ m/s. This led to an increase in the Darcy velocity of fracture water. When considering the stress field, the bedrock near the fracture wall would shrink due to the thermal contraction properties of the rock minerals with the injection of cold water. This leads to an increase of tensile stress near the fracture wall, which would increase fracture aperture and in turn lead to a small increase in Darcy velocity [24]. Ma et al. indicates that with the continuous injection of cold water, it gets colder and colder in the vicinity of the injection well over time [28]. This makes the water become more viscous, so the inlet flow rate becomes smaller. Our results are in agreement with the experiment results of Ref. [28]. It also proves the accuracy of the current modeling.

**Fig 7:**
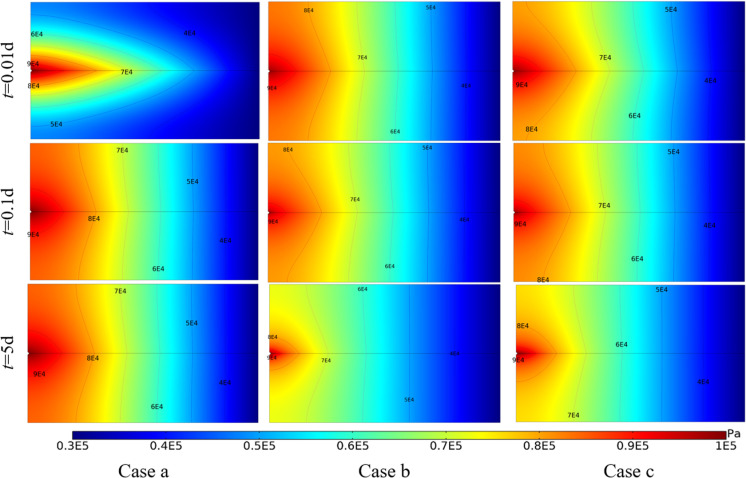
Variation of seepage pressure field with time under different cases.

Fig 7 shows the variation of seepage pressure field with time under different cases. In Case a, the hydraulic conductivity of the fracture is strong in the initial stage, and the seepage pressure in the fracture rises relatively fast. Then the seepage pressure in the bedrock gradually increases, and finally the whole seepage field reaches stability. Compared with Case a, the seepage pressure field in the initial stage of Case b and Case c spread to the whole reservoir faster. However, since the temperature field is constantly changing, the seepage pressure field is also constantly changing. The isobar moves towards the injection well with time. This is because the low temperature zone near the injection well expands over time. In addition, when the thermal stress is considered, the seepage pressure field near the injection well will increase further.

### Variation of temperature field

**Fig 8:**
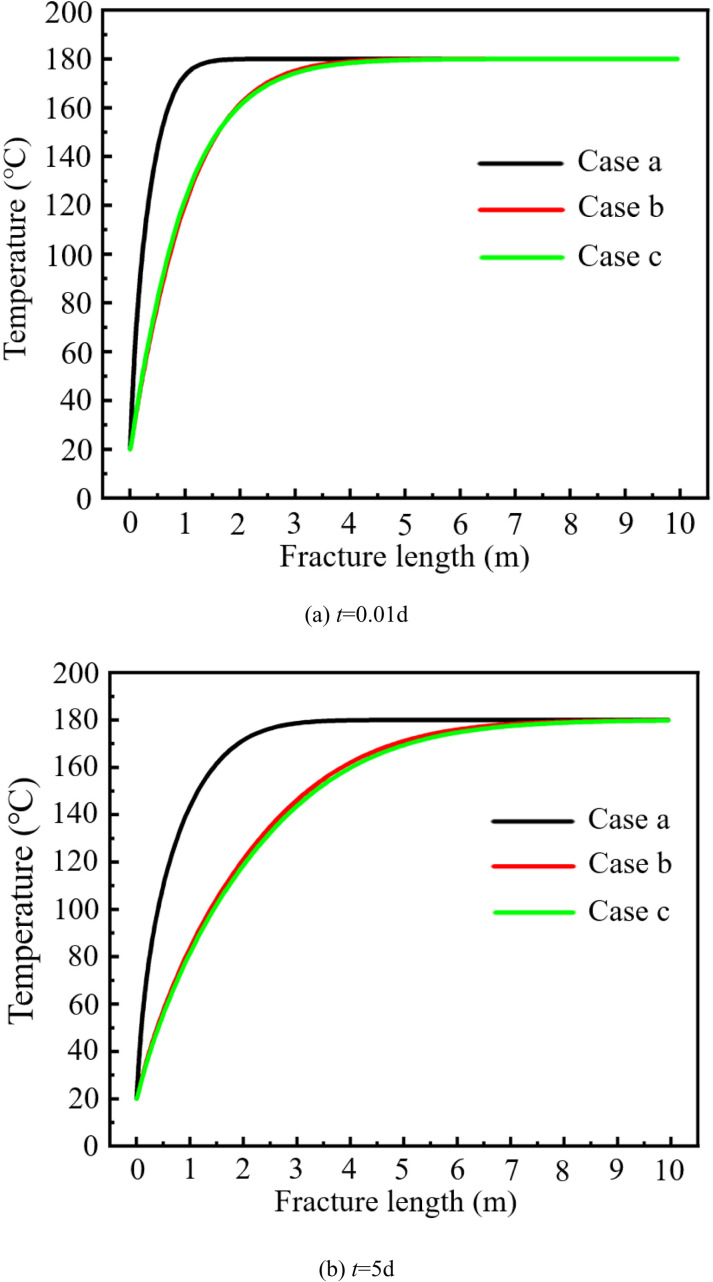
Temperature variation of fracture water with time under different cases.

Fig 8 shows the temperature variation of fracture water with time under different cases. In areas closer to the injection well, the temperature of fracture water rises dramatically. At the time node of *t* = 0.01d; the temperatures of the fracture water in Case a, Case b and Case c reach 180°C at *x* =  1.65m, 4.48m and 4.95m, respectively. At the time node of *t* = 5d; the temperatures of the fracture water in Case a, Case b and Case c reach 180°C at *x* =  3.48m, 8.37m and 9.06m, respectively.

Both the change of water property and the stress field will result in a larger Darcy velocity for the fracture water, which in turn will result in a larger distance for the water to reach a temperature of 180°C. When considering the change of water property, the distance for the fracture water to reach 180°C becomes rapidly longer. In contrast, when both the water property and the stress field are considered, the distance for the fracture water to reach 180°C is slightly greater than for the case where only the water property is considered. Comparing Fig 7 and Fig 8, it shows that the rapid change in temperature caused a change of water property which in turn led to a drastic increase in the flow rate at the entrance of the fracture.

**Fig 9:**
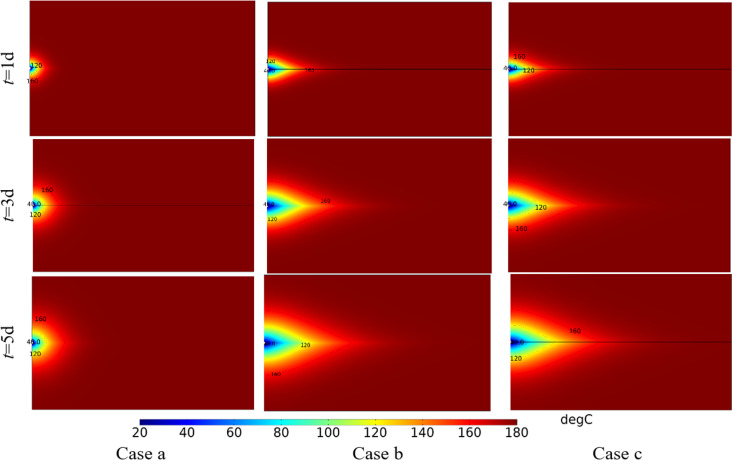
Variation of temperature field with time under different cases.

Fig 9 shows the variation of temperature field with time under different cases. With the continuous injection of cold water, the low temperature zone continues to expand from the injection well to the production well. Under Case a, the temperature near the injection well drops rapidly, forming a low temperature zone of approximately concentric ellipse.In the case of Case b and Case c, along the fracture direction, the temperature drops rapidly, forming a low temperature zone of approximately concentric cone. When considering the change of water property and stress field, the temperature field of surrounding rock would drop faster.

### Variation of stress field

**Fig 10:**
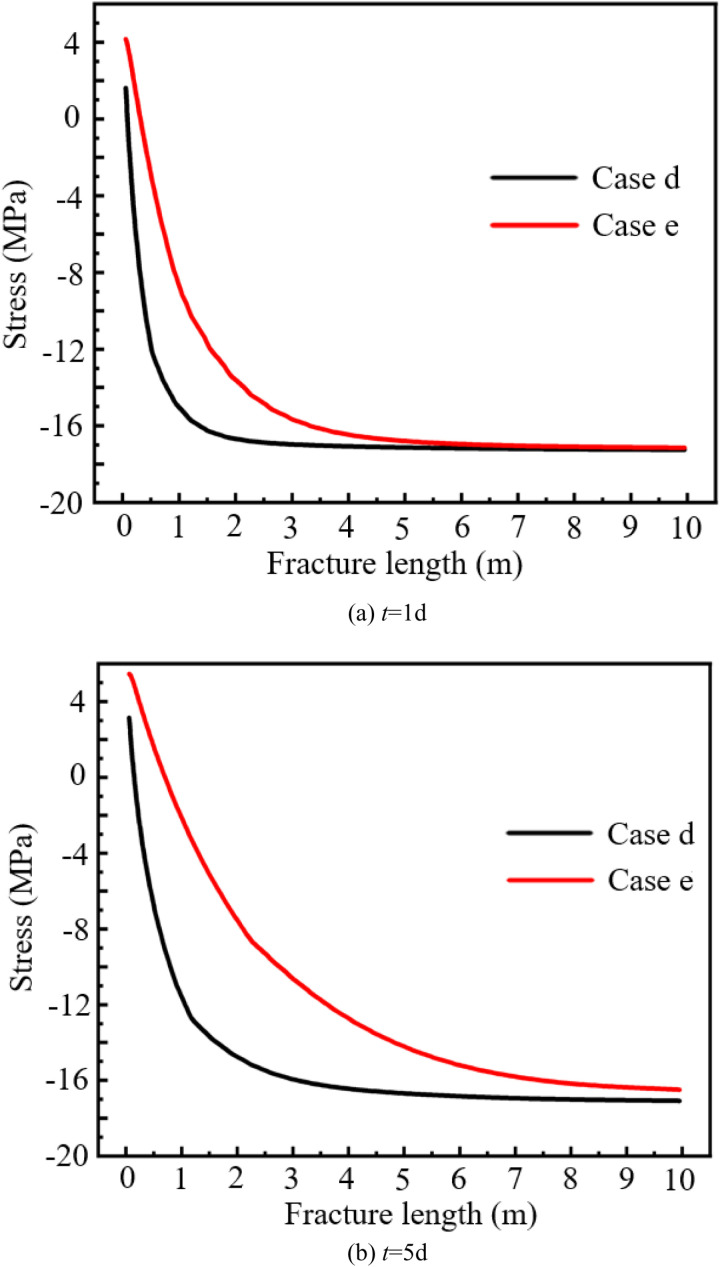
Stress distribution along the fracture wall under different cases.

From above research results, it can be seen that whether the change of water property is considered in the model has a great impact on the results. Therefore, the stress field distribution under two conditions without considering the change of water property with temperature (Case d) and considering the change of water property with temperature (Case e) is compared. Fig 10 shows the stress distribution along the fracture wall. The tensile stress is positive and the compressive stress is negative. When the stress changes from positive to negative, it indicates that the rock of geothermal reservoir shrinks and tensile stress is generated in the rock. Before the injection of cold water, compressive stress is distributed within the whole stress field. Due to the injection of cold water, the temperature near the fracture wall decreased rapidly, and the temperature difference of hundreds of degrees causes tensile stress in the rock. The tensile stress on the fracture wall decreases rapidly with the distance from the injection well.

At the time node of *t* = 1d; the maximum tensile stress along the fracture wall in Case d and Case e is 1.62MPa and 4.16MPa, respectively. At the time node of *t* = 5d; the maximum tensile stress along the fracture wall in Case d and Case e is 3.16MPa and 5.46MPa, respectively. If taking the tensile strength (5.25MPa) of granite measured in Ref. [28] as the criterion for rock fracturing, it can be seen that rock fracturing will be caused by tensile stress in Case e. Secondary micro-fractures will be generated in the direction perpendicular to the main fracture.

**Fig 11:**
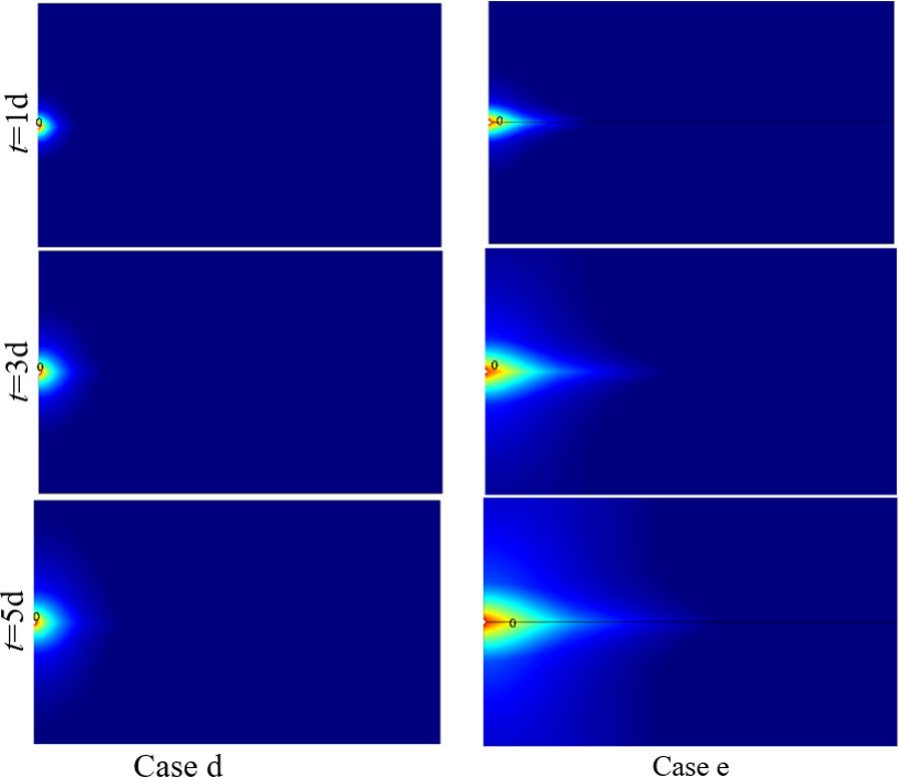
Variation of stress field with time under different cases.

Fig 11 shows the variation of stress field with time under different cases. With the continuous injection of cold water, the area of tension stress becomes larger and larger. Under the same time node, the tensile stress region of Case e is larger than that of Case d. At the time node of *t* = 1d; the positions of stress of 0 MPa under Case d and Case e along the fracture wall are 0.084m and 0.310m, respectively. At the time node of *t* = 5d; the positions of stress of 0 MPa under Case d and Case e along the fracture wall are 0.150m and 0.700m, respectively. It indicated that in the EGS project, the tension stress concentration area would be formed near the injection well, which may lead to the disaster of wellbore instability.

### Influence of DFN characteristics on the performance of EGS

#### DFN generation.

Referring to Refs. [29,30], the Monte Carlo method is used to generate random DFN. The location of the fracture is determined from the horizontal and vertical coordinates of the midpoint of the fracture obeying a uniform distribution. The fracture aperture is also set to obey a uniform distribution. The fracture trace length obeys a power law distribution. Its cumulative probability density function is shown


$$\begin{array}{c} L={\left(L_{min}^{-D}+F\left(L_{max}^{-D}-L_{min}^{-D}\right)\right)}^{-\frac{1}{D}} \end{array}$$ (24)

where *L* is the stitch trace length. *L*_*max*_ and *L*_*min*_ are the maximum and minimum fracture trace lengths, respectively. *F* is a uniformly distributed random number between 0 and 1. *D* is the fractal dimension.

**Fig 12:**
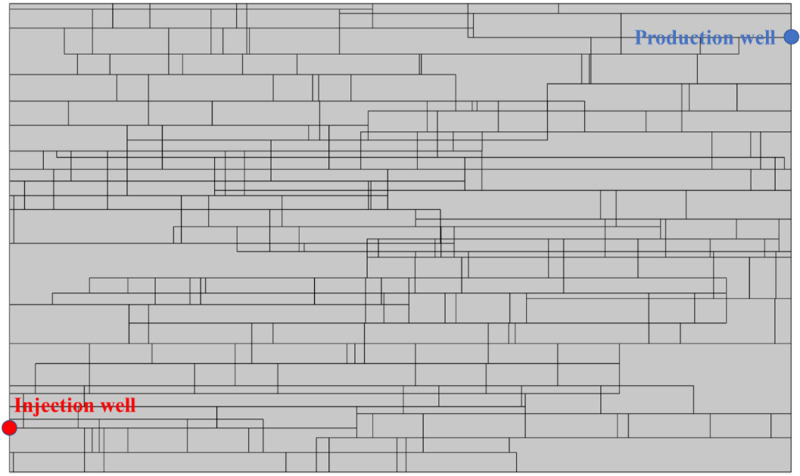
Randomly generated DFN with high horizontal in-situ stress.

MATLAB software is used for generating random DFN. Then it was imported into the COMSOL software. In addition, the DFN length and strike distributions are actually influenced by the nearby in-situ stress. Fig 12 shows a randomly generated DFN. It representing the horizontal in-situ stress being greater than the vertical in-situ stress, which accords with the state of horizontal stress regime in most deep EGS reservoir [31–33]. This generated DFN is used in this study. Horizontal fracture aperture is between 0.05 ~ 0.25 mm and vertical fracture opening is between 0.005 ~ 0.1 mm.

#### Model parameters, initial conditions and boundary conditions.

The established DFN model is a two-dimensional rectangle with 200 m long and 120 m wide. The locations of injection and production wells are shown in Fig 12. The model parameters are the same with that of above single fracture (seen in Table 1). Table 2 shows the model boundary conditions and initial conditions.

**Table 2:** Model boundary conditions and initial conditions.

Type	Seepage field	Temperature field	Stress field
Bedrock	2MPa	180℃	/
Injection well	8MPa	20℃	Fixed boundary
Production well	2MPa	Thermal outflow	Fixed boundary
Other borders	No flow exchange	Thermal insulation	Fixed boundary

#### Simulation schemes.

DFN is the main mass and heat transfer channel in EGS reservoir. In this paper, the influence of fracture density, fracture aperture, angle between fracture and well connection line and fracture pattern on the heat transfer performance of EGS is investigated with above THM coupled model. Production velocity, produced water temperature, equivalent permeability and thermal recovery efficiency were used to evaluate the thermal performance of EGS reservoir. The equivalent permeability could effectively characterize the permeability of the whole DFN reservoir. Thermal recovery efficiency is an important indicator for evaluating the operational life of EGS project. The equivalent permeability and thermal recovery efficiency are calculated by [34]:


$$\begin{array}{c} k_{\text{e}}=\frac{\bar{v}\mu L}{p_{\text{in}}-p_{\text{out}}} \end{array}$$ (25)

$$\begin{array}{c} \gamma =\frac{\iint \left[T_{0}-T\left(t\right)\right]dS}{\iint (T_{0}-T_{\text{inj}})dS} \end{array}$$ (26)

where *k*_e_ represents the equivalent permeability, m^2^ $.\bar{v}$ represents the average flow rate of the extraction well, m/s. *μ* represents the dynamic viscosity of water, Pas. *L* represents the distance between the injection well and the production well, m. *p*_in_ and *p*_out_ denote the injection and production pressures, Pa, respectively. *γ* represents the thermal recovery efficiency. *T*_0_ represents the initial temperature of the thermal storage, ℃. *T*(t) represents the temperature inside the reservoir at any moment, ℃. *T*_inj_ represents the temperature of the injected cold water, ℃.

The average temperature of the produced water is calculated by


$$\begin{array}{c} T_{\text{out}}=\frac{\sum u_{\text{frac}}d_{\text{frac}}T_{\text{frac}}+\int uTdy}{\sum u_{\text{frac}}d_{\text{frac}}+\int udy} \end{array}$$ (27)

where *u* is the flow rate, m/s. *d* is the fracture aperture, m. *T* is the temperature, °C. The subscript *frac* represents the fracture, and those without subscripts indicate bedrock.

## Results and discussions

### Fracture density

**Fig 13:**
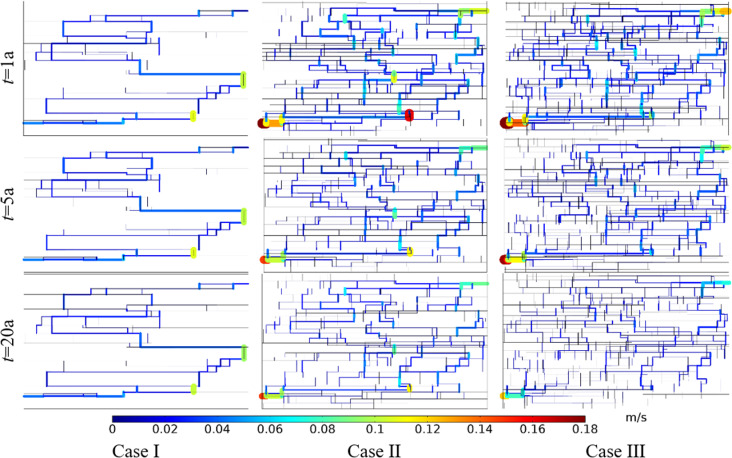
Comparison of Darcy’s velocity within the DFN for different fracture densities.

Taking the DNF system in Fig 12 as the basis (Case II), reducing the fracture number by 50% in both the *x* and *y* directions is taken as Case I, and increasing by 50% in both directions is taken as Case III. Fig 13 shows the comparison of Darcy’s velocity within the DFN system for different fracture densities. The production Darcy’s velocity in Case I, II and III in the first year were 0.009 m/s, 0.035 m/s and 0.041 m/s, respectively. The production Darcy’s velocity in Case I, II and III in the 5th year were 0.008 m/s, 0.029 m/s and 0.034 m/s, respectively. The production Darcy’s velocity in Case I, II and III in the 20th year were 0.008 m/s, 0.020 m/s and 0.024 m/s, respectively. It can be seen that with the increase of fracture density, the production Darcy’s velocity increases gradually, but the velocity increment gradually decreases with the operation. This is because the area of low temperature is expanding over time. Therefore, increasing the fracture density is conducive to increasing the production velocity in the stimulation of EGS reservoir.

The equivalent permeability in the first year under Case I, II and III were 2.86 ×  10^-10^ m^2^, 1.18 ×  10^-9^ m^2^ and 1.38 ×  10^-9^ m^2^, respectively. The equivalent permeability in the 5th year under Case I, II and III were 2.83 ×  10^-10^ m^2^, 9.76 ×  10^-10^ m^2^, and 1.14 ×  10^-9^ m^2^ respectively. The equivalent permeability in the 20th year under Case I, II and III were 2.69 ×  10^-10^ m^2^, 6.73 ×  10^-10^ m^2^ and 8.08 ×  10^-10^ m^2^, respectively. Increasing the fracture density could improve the connectivity of the fracture network system. However, when the fracture connectivity is enough good, the equivalent permeability increases smaller even though increasing the fracture density by further.

**Fig 14:**
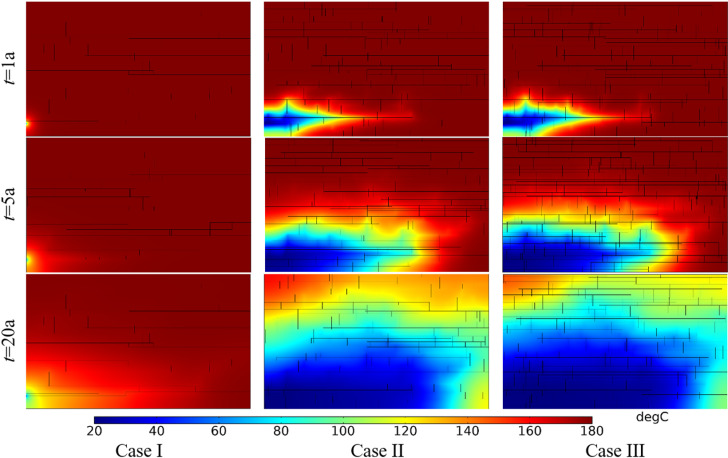
Comparison of temperature field within the DFN for different fracture densities.

Fig 14 shows the comparison of temperature field within the DFN for different fracture densities. The produced water temperature in Case I, II and III in the first year were 180.00°C, 180.00°C, and 180.00°C, respectively. The produced water temperature in Case I, II and III in the 5th year were 180.00°C, 179.91°C, and 179.85°C, respectively. The produced water temperature in Case I, II and III in the 20th year were 179.88°C, 135.58°C, and 118.52°C, respectively. For the same fracture density, the low-temperature zone increases with time. At the same time point, the low-temperature zone increases with fracture density. Increasing the fracture density can make the heat energy in the distant bedrock transfer more quickly to the main seepage fracture.

The thermal recovery efficiencies in Case I, II and III in the first year were 0.12%, 4.82% and 5.67%, respectively. The thermal recovery efficiencies in Case I, II and III in the 5th year were 0.75%, 20.42% and 24.11%, respectively. The thermal recovery efficiencies in Case I, II and III in the 20th year were 5.09%, 60.79% and 70.05%, respectively. The produced water temperatures and thermal recovery efficiencies of the three cases vary considerably. This is because the higher the fracture density, the better the connectivity of the DFN and the greater the seepage rate. This will take heat energy out of the thermal reservoir faster, so the difference in thermal recovery efficiency is significant.

### Fracture aperture

**Fig 15:**
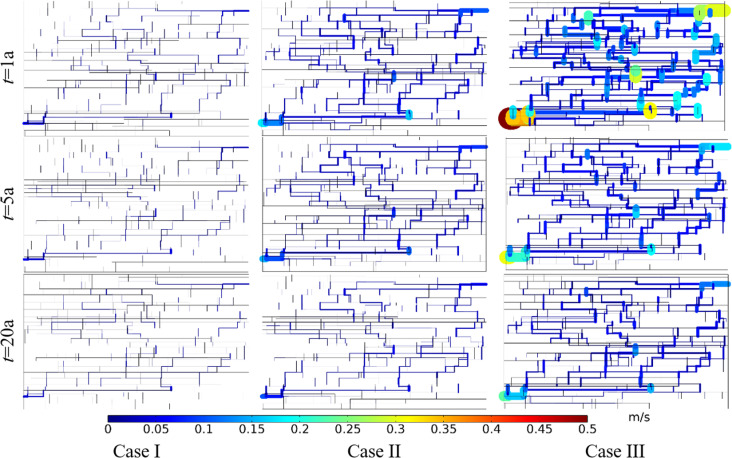
Comparison of Darcy’s velocity within the DFN for different fracture apertures.

Taking the DNF system in Fig 12 as the basis (Case II), reducing each fracture aperture by a factor of 1 is taken as Case I, and increasing by a factor of 1 is taken as Case III. Fig 15 shows the comparison of Darcy’s velocity within the DFN for different fracture apertures. The production Darcy’s velocity in Case I, II and III in the first year were 0.013 m/s, 0.035 m/s and 0.100 m/s, respectively. The production Darcy’s velocity in Case I, II and III in the 5th year were 0.011 m/s, 0.029 m/s and 0.059 m/s, respectively. The production Darcy’s velocity in Case I, II and III in the 20th year were 0.009 m/s, 0.020 m/s and 0.044 m/s, respectively. As the fracture aperture increases, the Darcy velocity within the DNF becomes larger overall. Increasing the fracture aperture can increase the permeability and conductivity of the DFN system.

The equivalent permeability in the first year under Case I, II and III were 4.38 ×  10^-10^ m^2^, 1.18 ×  10^-9^ m^2^ and 3.37 ×  10^-9^ m^2^, respectively. The equivalent permeability in the 5th year under Case I, II and III were 3.70 ×  10^-10^m^2^, 9.76 ×  10^-10^ m^2^ and 1.99 ×  10^-9^ m^2^, respectively. The equivalent permeability in the 20th year under Case I, II and III were 3.03 ×  10-^10^ m^2^, 6.73 ×  10^-10^ m^2^ and 1.48 ×  10^-9^ m^2^, respectively. It can be seen that increasing the fracture aperture could improve the connectivity of the fracture network system.

**Fig 16:**
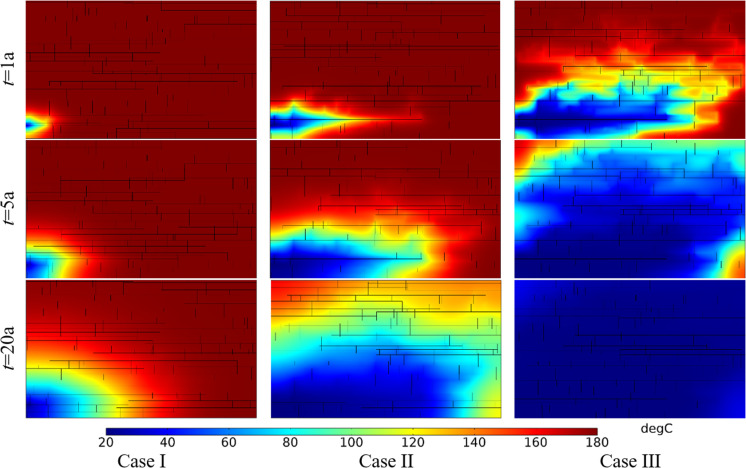
Comparison of temperature field within the DFN for different fracture apertures.

Fig 16 shows the comparison of temperature field within the DFN for different fracture apertures. The produced water temperature in Case I, II and III in the first year were 180.00°C, 180.00°C, and 177.44°C, respectively. The produced water temperature in Case I, II and III in the 5th year were 180.00°C, 179.91°C and 78.74°C, respectively. The produced water temperature in Case I, II and III in the 20th year were 179.96°C, 135.58°C and 22.62°C, respectively. At the same time point, the low-temperature zone increases with fracture aperture. In addition, compared with Fig 14, it can be seen that fracture aperture has a greater influence on the temperature field of the whole DFN reservoir than fracture density.

The thermal recovery efficiencies in Case I, II and III in the first year were 0.88%, 4.82% and 28.48%, respectively. The thermal recovery efficiencies in Case I, II and III in the 5th year were 3.78%, 20.42% and 80.75%, respectively. The thermal recovery efficiencies in Case I, II and III in the 20th year were 12.90%, 60.79% and 98.72%, respectively. The thermal recovery efficiency of the three cases varies considerably. This is because the larger the fracture aperture, the stronger the conductivity of the fracture, which would make the seepage rate greater. It will take away the heat energy from the heat reservoir faster.

### Angle between fracture and well connection line

**Fig 17:**
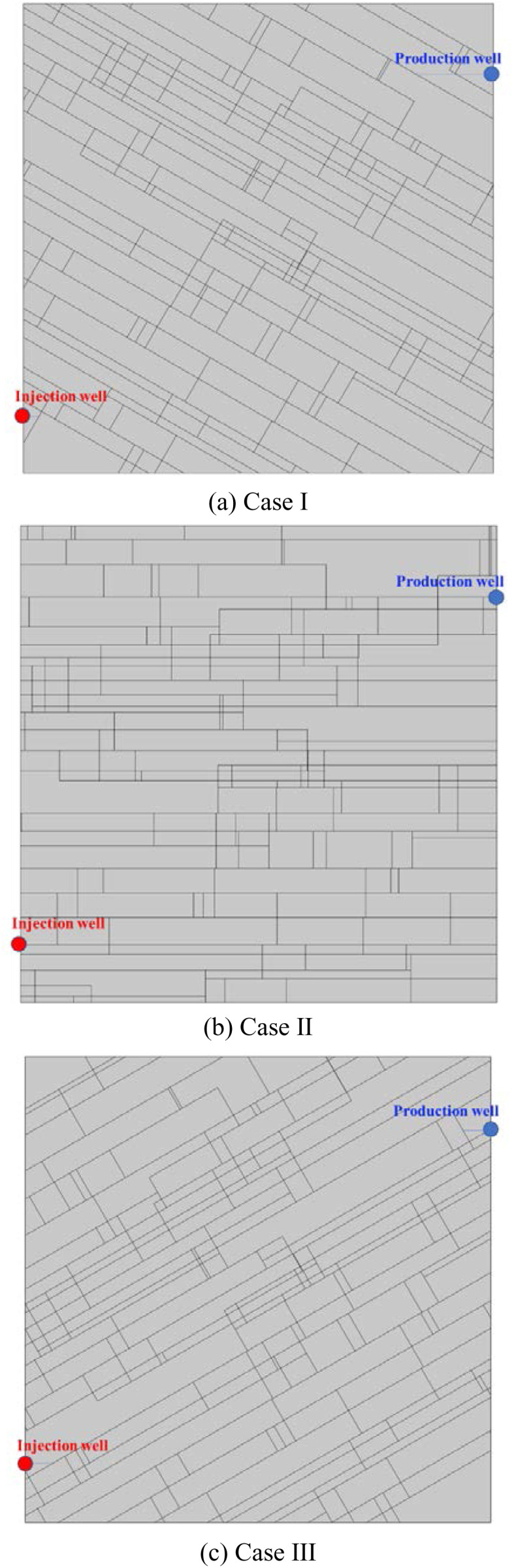
Three cases under different angles between fracture and well connection line.

The seepage system of EGS mainly includes injection well, production well and reservoir DFN system. Affected by the regional stress regime, the reservoir DFN system generally spreads along the direction of the regional maximum principal stress. The intersection angle of the injection-production well line and the main direction of the DFN system has an influence on the seepage and heat transfer of the EGS. Case I and Case III are obtained by rotating Case II by -30° and + 30°, respectively (Fig 17). The relative positions of injection well and production well remain unchanged in the three cases.

**Fig 18:**
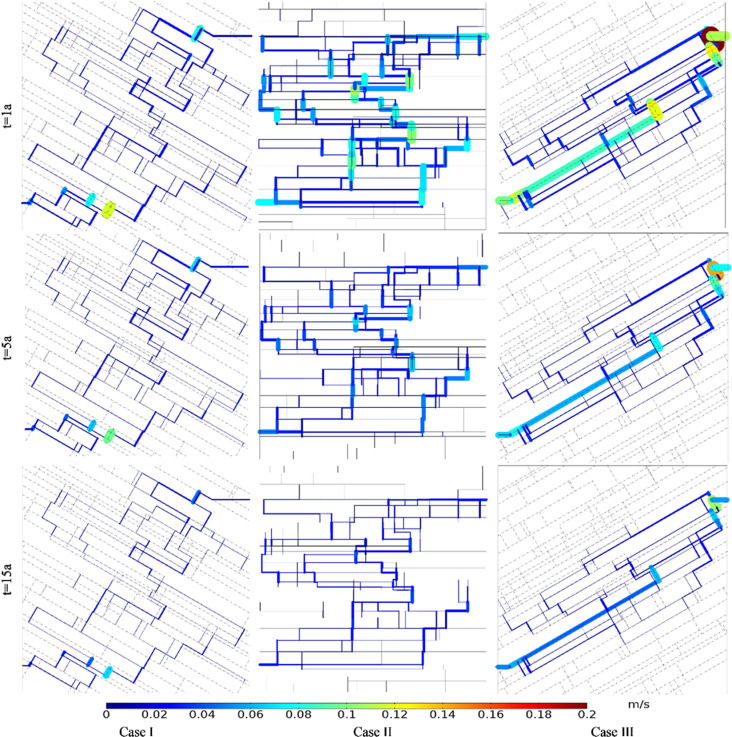
Comparison of Darcy’s velocity under different angles between fracture and well connection line.

Fig 18 shows the comparison of Darcy’s velocity under different angles between fracture and well connection line. The production Darcy’s velocity in Case I, II and III in the first year were 0.008 m/s, 0.028 m/s and 0.037 m/s, respectively. The production Darcy’s velocity in Case I, II and III in the 5th year were 0.007 m/s, 0.018 m/s and 0.025 m/s, respectively. The production Darcy’s velocity in Case I, II and III in the 15th year were 0.005 m/s, 0.011 m/s and 0.019 m/s, respectively. At the same time point, the flow net area of Case I and Case II is obviously larger than that of Case III, but the Darcy’s velocity in Case I is obviously smaller than that of Case II and Case III. It can be seen that the smaller the angle between fracture and well connection line, the more favorable it is for the seepage of the working fluid.

The equivalent permeability in the first year under Case I, II and III were 5.39 ×  10^-10^ m^2^, 1.87 ×  10^-9^ m^2^ and 2.46 ×  10^-9^ m^2^, respectively. The equivalent permeability in the 5th year under Case I, II and III were 4.44 ×  10^-10^ m^2^, 1.23 ×  10^-9^ m^2^ and 1.68 ×  10^-9^ m^2^, respectively. The equivalent permeability in the 15th year under Case I, II and III were 3.37 ×  10^-10^ m^2^, 7.41 ×  10^-10^ m^2^ and 1.28 ×  10^-9^ m^2^, respectively. When the hydraulic connectivity of the DFN system remains unchanged, the smaller the angle between fracture and well connection line, the smaller the flow resistance of the working fluid, and the larger the equivalent permeability of the reservoir DFN system.

**Fig 19:**
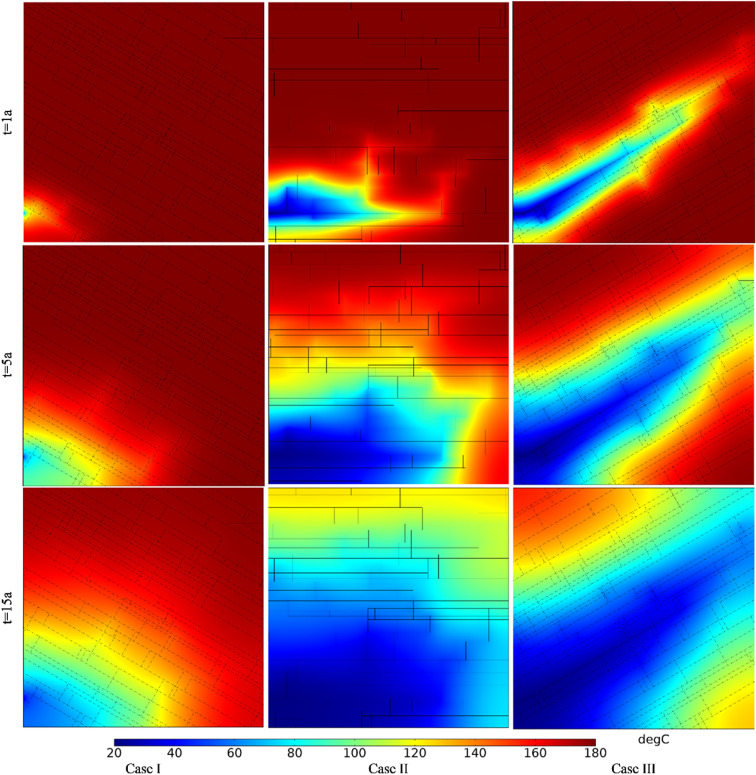
Comparison of temperature field under different angles between fracture and well connection line.

Fig 19 shows the comparison of temperature field under different angles between fracture and well connection line. The produced water temperature in Case I, II and III in the first year were 180.00°C, 180.00°C and 167.88°C, respectively. The produced water temperature in Case I, II and III in the 5th year were 180.00°C, 171.85°C and 109.12°C, respectively. The produced water temperature in Case I, II and III in the 15th year were 178.34°C, 112.10°C and 66.78°C, respectively. Thermal breakthrough time of production wells in Case Ⅱ and Case Ⅲ is earlier than that in Case Ⅰ. The temperature decreasing rate of production well in Case Ⅲ is greater than that in Case Ⅱ. The reasons for the premature thermal breakthrough in Case Ⅲ is that the seepage rate in Case Ⅲ is high and dominant seepage channel is formed with a small flow net area. At the same time node, the extent of the low-temperature zone in Case I is smaller than that in Cases II and III. The extent of the low-temperature zone in Case III is narrower and longer than that in Case II. Therefore, the smaller the angle between fracture and well connection line, the faster the low-temperature zone evolves along the direction of well connection line.

The thermal recovery efficiencies in Case I, II and III in the first year were 0.84%, 9.04% and 12.90%, respectively. The thermal recovery efficiencies in Case I, II and III in the 5th year were 6.14%, 35.83% and 38.11%, respectively. The thermal recovery efficiencies in Case I, II and III in the 15th year were 19.29%, 68.28% and 64.00%, respectively. During the first five years, the smaller the angle between fracture and well connection line, the greater the thermal recovery efficiency. However, with the continuous water circulation, the thermal recovery efficiency of Case II has exceeded that of Case III by the 15th year. The reason may be that the heat energy in the surrounding rock on both sides of the dominant seepage channel is mainly extracted in Case III. However, in the late phase of Case II, the seepage through the DFN range is wider than that of Case III. It can extract more heat from larger reservoirs. Therefore, from the point of the thermal recovery efficiency, the EGS project should make the connection between the fracture and the well line at a certain acute angle.

### DFN pattern

**Fig 20:**
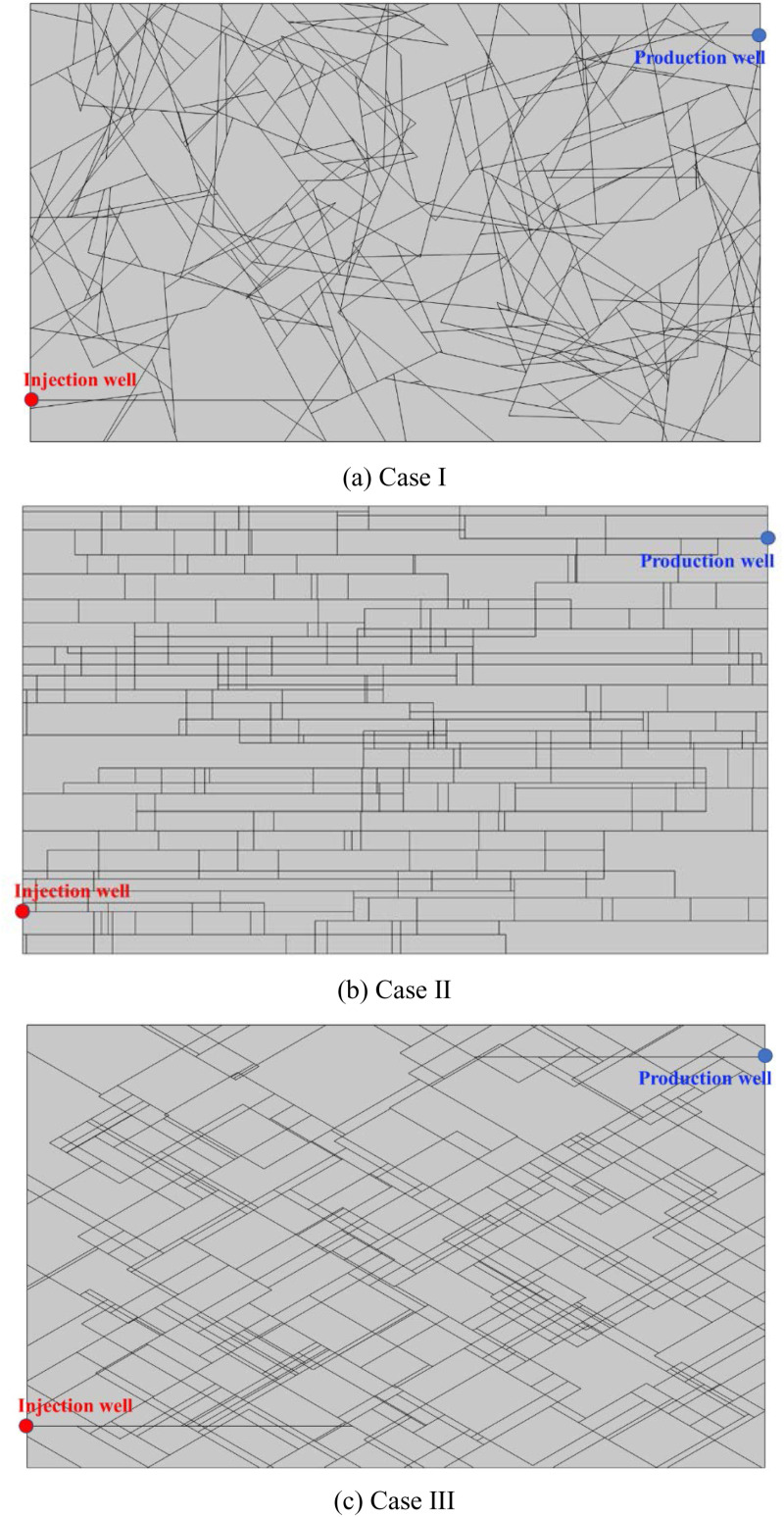
The generated three DFN patterns.

In the long geological history, the deep strata may have formed different DFN patterns due to many periods of thrust and tension action. In the selection of EGS reservoir, the optimum DFN pattern should be selected according to the thermal performance analysis. When studying the influence of DFN pattern on thermal performance of EGS, it is necessary to ensure the consistency of random fracture distribution feature function and value range. In this study, three fracture network distribution cases are generated with random direction (Case I), fixed direction (0° +  90°, Case II), and fixed direction (30° +  150°, Case III). In order to ensure that the relative positions of the injection and production well remain unchanged, the fractures where the wells are located in Case Ⅱ are retained in Case Ⅰ and Case Ⅲ. Table 3 listed the parameters of three DFN patterns. Fig 20 shows the generated three DFN patterns.

**Table 3:** Parameters of three DFN patterns.

Pattern	Fracture direction	Fracture number	Fracture aperture
Case I	Random	Group 1: 40	0.05-0.25 mm
		Group 2: 200	0.005-0.1 mm
Case II	Fixed (0° + 90°)	Horizontal: 40	0.05-0.25 mm
		Vertical: 200	0.005-0.1 mm
Case III	Fixed (30° + 150°)	Group 1: 20 (30°) + 20(150°)	0.05-0.25 mm
		Group 2: 100 (30°) + 100(150°)	0.005-0.1 mm

**Fig 21:**
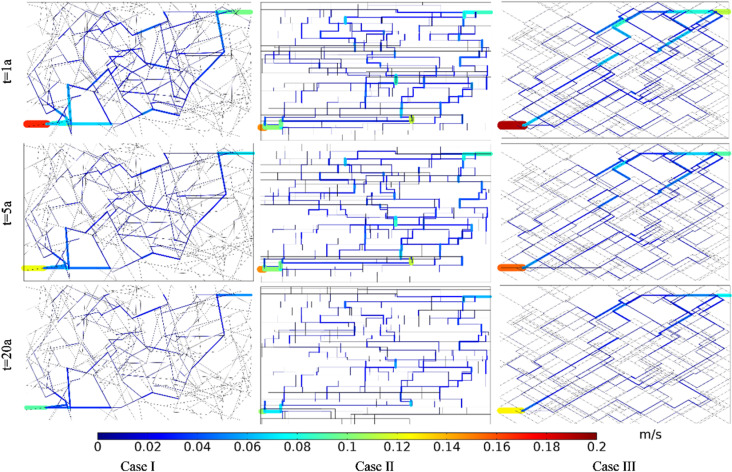
Comparison of Darcy’s velocity under different DFN patterns.

Fig 21 shows the comparison of Darcy’s velocity under different DFN patterns. The fracture water flows from injection well to production well according to their respective optimal paths in the three cases. The production Darcy’s velocity in Case I, II and III in the first year were 0.033 m/s, 0.035 m/s and 0.037 m/s, respectively. The production Darcy’s velocity in Case I, II and III in the 5th year were 0.024 m/s, 0.029 m/s and 0.031 m/s, respectively. The production Darcy’s velocity in Case I, II and III in the 20th year were 0.018 m/s, 0.020 m/s and 0.022 m/s, respectively. The production Darcy’s velocity from the production well in the three cases are very close to each other. Therefore, although the DFN distribution varies greatly, the differences in the production Darcy’s velocity are small for the same range of statistical characteristics and parameter values of the DFN patterns.

The equivalent permeability in the first year under Case I, II and III were 1.11 ×  10^-9^ m^2^, 1.18 ×  10^-9^ m^2^ and 1.25 ×  10^-9^ m^2^, respectively. The equivalent permeability in the 5th year under Case I, II and III were 8.08 ×  10^-10^ m^2^, 9.76 ×  10^-10^ m^2^ and 1.04 ×  10^-9^ m^2^, respectively. The equivalent permeability in the 20th year under Case I, II and III were 6.06 ×  10^-10^ m^2^, 6.73 ×  10^-10^ m^2^ and 7.41 ×  10^-10^ m^2^, respectively. The equivalent permeability of three cases is very similar for the same range of statistical characteristics and parameter values of the DFN patterns.

**Fig 22:**
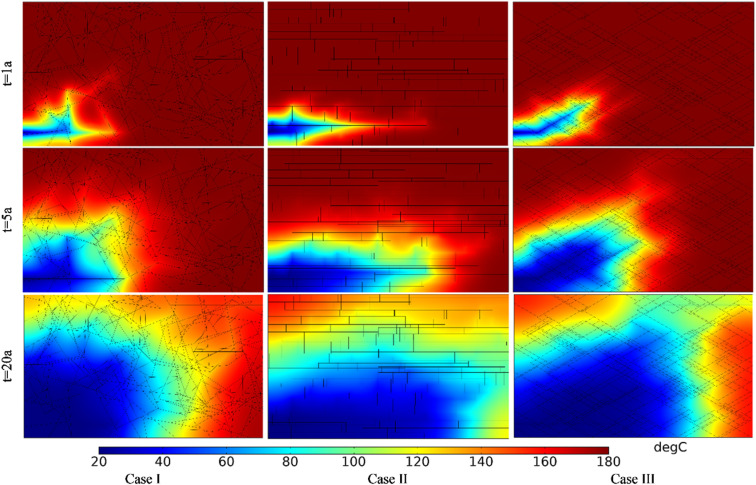
Comparison of temperature field distribution under different DFN patterns.

Fig 22 shows the comparison of temperature field distribution under different DFN patterns. The produced water temperature in Case I, II and III in the first year were 180.00°C, 180.00°C and 180.00°C, respectively. The produced water temperature in Case I, II and III in the 5th year were 179.55°C, 179.91°C and 178.31°C, respectively. The produced water temperature in Case I, II and III in the 20th year were 152.99°C, 135.58°C and 117.05°C, respectively. Thermal breakthrough time of production well in Case Ⅱ and Case Ⅲ is earlier than that in Case Ⅰ. The temperature decreasing rate of production well in Case III is higher than that in Case I and Case II. Although the morphology of the low-temperature zone is quite different, the evolution direction of the low-temperature zone is consistent with the direction of the dominant seepage flow in the fracture.

The thermal recovery efficiencies in Case I, II and III in the first year were 4.59%, 4.82% and 5.04%, respectively. The thermal recovery efficiencies in Case I, II and III in the 5th year were 17.70%, 20.42% and 21.48%, respectively. The thermal recovery efficiencies in Case I, II and III in the 20th year were 52.52%, 60.79% and 60.83%, respectively. At the initial stage of EGS project operation, the thermal recovery efficiency of different DFN systems is not different. With the continuous operation of the project, the thermal recovery efficiency of Case II and Case III become relatively close to each other and slightly greater than that of Case I. Compared with the results of the seepage field in Fig 21, it can be seen that even though the velocity difference under different fracture patterns is small, with the perennial operation of the project, this seepage velocity difference will have a great impact on the produced water temperature and reservoir thermal performance. In addition, considering the production water temperature and thermal recovery efficiency comprehensively, the DFN systems of Case II and Case I is better than that of Case III. Therefore, the DFN system with uniform fracture distribution should be selected for a better EGS reservoir, and the angle between the direction of the main fracture group and the direction of the well connection line should be avoided too small.

## Conclusions

In this paper, a coupled THM multi-physical field model of EGS is established and proved with the analytical solution. The variation of water density, thermal conductivity and specific heat capacity with temperature is considered. The influence of tension stress caused by temperature difference between injected cold water and bedrock is discussed. The effect of DFN characteristics on thermal performance of EGS was numerically investigated. The following conclusions were obtained:

(1) The effect of considering the change of water property on the Darcy velocity is larger than considering the stress field. Considering the change of water property and considering the stress field both lead to a larger Darcy velocity of the fracture water. When considering the stress field, the bedrock near the fracture wall would shrink due to the thermal contraction properties of the rock minerals with the injection of cold water. This leads to an increase of tensile stress near the fracture, which would increase fracture aperture and in turn lead to an increase in Darcy velocity. When considering the change of water property and stress field, the temperature field of surrounding rock drops faster. In the EGS project, the tension stress concentration area will be formed near the injection well, which may generate multiple sets of new fractures.
(2) The influence of fracture density, fracture aperture, angle between fracture and well connection line and fracture pattern on the heat transfer performance of EGS is investigated with the coupled THM model. Production Darcy’s velocity, produced water temperature, equivalent permeability and thermal recovery efficiency, were used to evaluate the thermal performance of EGS reservoir.
(3) With the increase of fracture density, the production Darcy’s velocity increases gradually, but the increment will gradually decrease with time. Increasing the fracture density could improve the connectivity of the fracture network system. However, when the fracture connectivity is enough good, the equivalent permeability increases smaller by further increasing the fracture density. The low-temperature zone increases with fracture density. Increasing the fracture density can make the heat energy in the distant bedrock transfer more quickly to the main seepage fracture. The higher the fracture density, the better the connectivity of the DFN and the greater the seepage rate. This will take heat energy out of the thermal reservoir faster, so the difference in thermal recovery efficiency is significant.
(4) As the fracture aperture increases, the Darcy velocity within the DNF becomes larger overall. Increasing the fracture aperture can increase the permeability and conductivity of the DFN system. Fracture aperture has a greater influence on the temperature field of the whole DFN reservoir than fracture density. The larger the fracture aperture, it will take away the heat energy from the heat reservoir faster. The low-temperature zone area increases with the increase of fracture aperture.
(5) When the hydraulic connectivity of the DFN system remains unchanged, the smaller the angle between fracture and well connection line, the smaller the flow resistance of the working fluid, and the larger the equivalent permeability of the reservoir DFN system. During the first five years, the smaller the angle between fracture and well connection line, the greater the thermal recovery efficiency. However, with the continuous water circulation, the thermal recovery efficiency may change between different cases. Therefore, from the point of thermal recovery efficiency, the EGS project should make the connection between the fracture and the well line at a certain acute angle.
(6) Although the DFN distribution varies greatly, the differences in the production Darcy’s velocity and equivalent permeability are small for the same range of statistical characteristics and parameter values of the DFN patterns. The evolution direction of the low-temperature zone is consistent with the direction of the dominant seepage flow in the fracture. At the initial stage of EGS project operation, the thermal recovery efficiency of different DFN systems is not different. With the continuous operation of the project, the thermal recovery efficiency of different DFN patterns become relatively close to each other. Even though the velocity difference under different fracture patterns is small, with the perennial operation of the project, this seepage velocity difference will have a great impact on the produced water temperature and reservoir thermal performance. The DFN system with uniform fracture distribution should be selected for a better EGS reservoir, and the angle between the direction of the main fracture group and the direction of the well connection line should be avoided too small.

## Supporting information

S1 Table Material parameters of granite used in the model. (DOCX)

S2 Table Model boundary conditions and initial conditions. (DOCX)

S3 Table Parameters of three DFN patterns. (DOCX)
