# Supplementary material for: Influence of discrete fracture network on the performance of enhanced geothermal system considering thermal-hydraulic-mechanical multi-physical field coupling
Source: PLoS One. 2025 Apr 23;20(4):e0320015. doi: 10.1371/journal.pone.0320015 (PMC12017569; doi:10.1371/journal.pone.0320015)
Supplement: S1 Table — (DOCX) [file pone.0320015.s001.docx]

**S1 Table. Material parameters of granite used in the model.**

| Parameters | Value |
| --- | --- |
| Density | 2.608 g/cm^3^ |
| Porosity | 1.46% |
| Permeability | 1.00 × 10^-18^ m^2^ |
| Thermal conductivity | 2.788 W/(m∙℃) |
| Specific heat capacity | 770 J/(kg∙℃) |
| Elastic modulus | 32.17 GPa |
| Poisson's ratio | 0.28 |
| Coefficient of thermal expansion | 3.60 × 10^-6^ ℃^-1^ |
