# Supplementary material for: Influence of discrete fracture network on the performance of enhanced geothermal system considering thermal-hydraulic-mechanical multi-physical field coupling
Source: PLoS One. 2025 Apr 23;20(4):e0320015. doi: 10.1371/journal.pone.0320015 (PMC12017569; doi:10.1371/journal.pone.0320015)
Supplement: S2 Table — (DOCX) [file pone.0320015.s002.docx]

**S2 Table. Model boundary conditions and initial conditions.**

| Type | Seepage field | Temperature field | Stress field |
| --- | --- | --- | --- |
| Bedrock | 2MPa | 180℃ | / |
| Injection well | 8MPa | 20℃ | fixed boundary |
| Production well | 2MPa | thermal outflow | fixed boundary |
| Other borders | no flow exchange | thermal insulation | fixed boundary |
