# Supplementary material for: Influence of discrete fracture network on the performance of enhanced geothermal system considering thermal-hydraulic-mechanical multi-physical field coupling
Source: PLoS One. 2025 Apr 23;20(4):e0320015. doi: 10.1371/journal.pone.0320015 (PMC12017569; doi:10.1371/journal.pone.0320015)
Supplement: S3 Table — (DOCX) [file pone.0320015.s003.docx]

**S3 Table. Parameters of three DFN patterns.**

| Pattern | Fracture direction | Fracture number | Fracture aperture |
| --- | --- | --- | --- |
| Case I | Random | Group 1: 40 | 0.05-0.25 mm |
|  |  | Group 2: 200 | 0.005-0.1 mm |
| Case II | Fixed (0° + 90°) | Horizontal: 40 | 0.05-0.25 mm |
|  |  | Vertical: 200 | 0.005-0.1 mm |
| Case III | Fixed (30° + 150°) | Group 1: 20 (30°)+20(150°) | 0.05-0.25 mm |
|  |  | Group 2: 100 (30°)+100(150°) | 0.005-0.1 mm |
